# A cost minimisation analysis in teledermatology: model-based approach

**DOI:** 10.1186/1472-6963-10-251

**Published:** 2010-08-25

**Authors:** Nina Eminović, Marcel G Dijkgraaf, Rosanne M Berghout, Astrid H Prins, Patrick JE Bindels, Nicolette F de Keizer

**Affiliations:** 1 Department of Medical Informatics, Academic Medical Center-University of Amsterdam, P. O. Box 22660, 1100 DD Amsterdam, The Netherlands; 2 Department of Clinical Epidemiology, Biostatistics and Bioinformatics, Academic Medical Center-University of Amsterdam, P. O. Box 22660, 1100 DD Amsterdam, The Netherlands; 3 Department of Facilities Management, University Medical Center Utrecht, Postbus 85500, 3508 GA Utrecht, The Netherlands; 4 Department of General Practice, Academic Medical Center-University of Amsterdam, P. O. Box 22660, 1100 DD Amsterdam, The Netherlands

## Abstract

**Background:**

Although store-and-forward teledermatology is increasingly becoming popular, evidence on its effects on efficiency and costs is lacking. The aim of this study, performed in addition to a clustered randomised trial, was to investigate to what extent and under which conditions store-and-forward teledermatology can reduce costs from a societal perspective.

**Methods:**

A cost minimisation study design (a model based approach) was applied to compare teledermatology and conventional process costs per dermatology patient care episode. Regarding the societal perspective, total mean costs of investment, general practitioner, dermatologists, out-of-pocket expenses and employer costs were calculated. Uncertainty analysis was performed using Monte Carlo simulation with 31 distributions in the used cost model. Scenario analysis was performed using one-way and two-way sensitivity analyses with the following variables: the patient travel distance to physician and dermatologist, the duration of teleconsultation activities, and the proportion of preventable consultations.

**Results:**

Total mean costs of teledermatology process were €387 (95%CI, 281 to 502.5), while the total mean costs of conventional process costs were €354.0 (95%CI, 228.0 to 484.0). The total mean difference between the processes was €32.5 (95%CI, -29.0 to 74.7). Savings by teledermatology can be achieved if the distance to a dermatologist is larger (> = 75 km) or when more consultations (> = 37%) can be prevented due to teledermatology.

**Conclusions:**

Teledermatology, when applied to all dermatology referrals, has a probability of 0.11 of being cost saving to society.

In order to achieve cost savings by teledermatology, teledermatology should be applied in only those cases with a reasonable probability that a live consultation can be prevented.

**Trail Registration:**

This study is performed partially based on PERFECT D Trial (Current Controlled Trials No.ISRCTN57478950).

## Background

Teledermatology can be defined as the use of imaging and telecommunication technologies to provide skin services by a dermatologist to another health professional or a patient [1]. Teledermatology can be useful to increase access to care in rural and remote areas [2], but can also potentially decrease the need for outpatient hospital consultations and, as a consequence, reduce societal costs of skin treatment.

Based on logistics and technical equipment, two types of teledermatology can be distinguished: real-time and store-and-forward (SAF). In real-time teledermatology synchronous communication is established using videoconferencing equipment. The SAF variant is less demanding due to its asynchronous communication by e-mail or a web-based application and therefore perhaps less costly. Hence, the popularity of SAF teledermatology is increasing, even in densely populated countries with small distances. Still, evidence of decreasing numbers of outpatient hospital referrals and cost savings due to teledermatology is currently lacking [1,3]. In particular, economic evaluations of SAF teledermatology services in comparison to conventional dermatological care are lacking [1,4].

We conducted a clustered randomised trial (*PERFECT D: Primary care Electronic Referrals: Focus on Efficient Consultation using Telemedicine in Dermatology*, trial registration: Current Controlled Trials No: ISRCTN57478950) to assess whether teledermatology, used as an electronic SAF referral system, could decrease the number of outpatient hospital referrals by the general practitioner (GP) [5]. In our teledermatology setting, the GP could ask the dermatologist advice on how to treat their dermatological patients. The main objective of the economic evaluation study described in this paper was to investigate to what extent and under which conditions SAF teledermatology can reduce the costs from a societal perspective.

## Methods

### Clustered RCT on teledermatology

The PERFECT D study was performed in the catchments areas of two Dutch general district hospitals in Almere and Zeist. Patients were eligible if they were referred by their GP to one of the recruited dermatologists, did not require an urgent consultation (within one or two days) and gave written informed consent. We randomised GPs in two groups: an intervention and a control group. Intervention group GPs took at least two, maximally four digital images of the skin problem and attached these to a semi-structured form which they completed on a secured website. We used KSYOS TDCS^® ^teledermatology website which was adjusted for the study (anonymous patient data) and a KODAK CX6230 digital camera. The dermatologist to whom the patient was assigned received a notification email and responded within two days using the same secured website. Based on digital images and semi-structured form, the dermatologist advised the GP about further procedures such as treatment, additional investigations, need for standard or urgent referral that GP could perform.

In control practices, patients were referred to a dermatologist following the usual care process. For the purpose of this study, all patients from the intervention and control group were required to visit a dermatologist in the outpatient hospital clinic and were placed on the regular waiting list (can take up to twelve weeks in some hospitals), irrespective of their degree of recovery. During this visit the dermatologist determined whether the live consultation was appropriate (e.g. the required intervention can only be performed by a dermatologist) or not (e.g. the patient was already recovered because of teledermatology or spontaneously). A consultation was also considered preventable if a dermatologist concluded a GP could also have treated a patient instead of referring the patient.

Eighty-five GPs and five dermatologists participated in the trial including 631 patients (intervention: 327, control: 304). Our trial showed that using teledermatology in general practice made 39% of consultations preventable, while this percentage was 18.3% in the control group. It was concluded that 20.7% of dermatological consultations were preventable due to teledermatology.

The ethics committee considered this study to be exempt from review because the research did not interfere with usual care. Details and the results from the clustered randomised trial (PERFECT D) have been described separately [5].

### Design of the economic evaluation

Teledermatology reorganizes the care process of skin patients by shifting responsibilities between primary and secondary care, but it does not imply a different, new treatment for these patients. Although teledermatology can lead to an earlier diagnosis and thereby earlier treatment, after a certain period patients are equally recovered with or without teledermatology. A recent study demonstrated that differences regarding clinical outcomes between conventional care and teledermatology care did not exist, which justified a cost minimisation study design [6,7].

The economic evaluation was performed following a decision analytical approach [8]. A model was built containing the volume and unit cost parameters of the teledermatology and conventional process. The time horizon was defined as the length of the dermatological episode of care, starting with the referral of the patient by a GP to a dermatologist and ending within six months after the referral. This time horizon was chosen as it was expected that within this period all dermatological patients would be equally recovered. This period was equal for the conventional and teledermatology process. In the trial, control and intervention patients (even when they were recovered) had similar waiting times up to six months to visit a dermatologist [5]. The study by Pak et al. [6] showed equal recovery after four months.

As our time horizon was less than a year, no discounting was applied.

**Figure 1:**
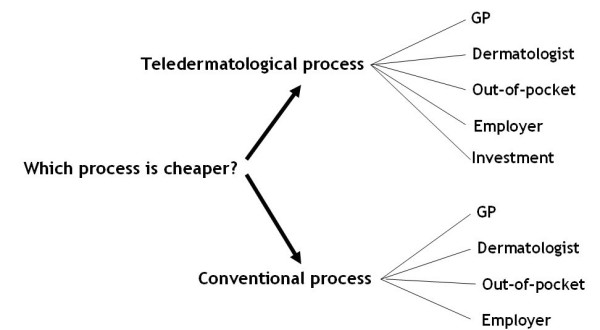
**Graphical representation of the model where the choice is made between the conventional and teledermatological process**. The key components of these processes are illustrated.

Five major costs components were distinguished: investments, GP care, dermatology care, out-of-pocket expenses by patients, and employer costs (see Figure 1 and Table 1). In total 282 variables were used in the model.

**Table 1:** Parameter estimates and related uncertainty for a selection of most important volume components and unit costs

	Value	Uncertainty analysis (UA)** Scenario analysis (SA)	Range	Distribution parameters (T: Triangular/ β: beta(integral form))	Source
**Investment costs**					

Digital camera price per 3 years	€ 175	UA	50 - 300	T (mode = 175)	Market prices

Website application development for 3 years	€ 40 k	UA	20 k - 70 k	T (mode = 40 k)	Expert opinion

Number of accounts using the service	5000	UA	2000 - 8000	T (mode = 5000)	Expert opinion

Duration training GP (minutes)	35	UA	10 - 50	T (mode = 35)	Trial observation

Duration training dermatologist (minutes)	20	UA	5 - 35	T (mode = 20)	Trial observation

Trainer hourly rate	€ 16.6	UA	10 - 25	T (mode = 16.6)	Expert opinion

**GP**					

Duration consultation (minutes)	8.1				Nivel [9]

Costs GP per minute	€ 2.5				Dutch Manual for Costing [11]

Duration teleconsultation activities (minutes)	11.6	UA, SA	4.6 - 28.8	This parameter consists of nine different parameters **^9^**	Trial data [10]

**Dermatologist**					

Duration consultation (minutes)	10			NA	Dutch Manual for Costing [11]

Costs dermatologist per minute	€ 5.6 (*academic*), € 6.7 (*general*)			NA	Dutch Manual for Costing [11]

Teleconsultation duration (minutes)	8.3	UA, SA	4.5 - 13.1	This parameter consists of three different parameters	Trial observation

Diagnostics and treatment	€ 87.6	UA	60 -115	Diagnosis T (mode = 50) Treatment (mode = 40)	Calculated based on prevalence information, guidelines, expert opinions and tariff books

Number of visits per episode	3	UA	1-5	T (mode = 3)	Expert opinion

**Out-of-pocket**					

Distance to GP (km)	1.8	SA	1 - 60	NA	Dutch Manual for Costing [11]

Distance to dermatologist (km)	7	SA	3 - 200	NA	Dutch Manual for Costing [11]

Travel costs per km, public transportation or car	€ 0.25		NA	NA	Dutch Manual for Costing [11]

Parking costs per visit	€ 2.5		NA	NA	Dutch Manual for Costing [11]

Proportion patients visiting a GP or dermatologist in company	0.2	UA	0-0.5	β (r = 2, n = 10)	Educated guess based on proportion of elderly and children

**Employer costs**					

Duration one visit GP (minutes) *	33.2	UA	25 - 40	Consists of several parameters*	Dutch Manual for Costing [11]+ estimation

Duration one visit dermatologist (minutes)*	71	UA	40 - 100	Consists of several parameters*	Dutch Manual for Costing [11]+ estimation

Costs per hour	€ 35	UA	10 - 50	T (mode = 35)	Based on average income in the Netherlands

**General**					

Proportion of preventable referrals	0.20	UA, SA	0.1 - 0.50	β (r = 4, n = 19)	Trial data [5]

*travel time, waiting time, consultation time, time picking up medication

** Other parameters included in the uncertainty analysis included: number of account teleconsultation system (T (min 2000, mode 5000, max 8000)) and parameters concerning the waiting time in the GP and dermatologist office.

The investments covered the costs of the digital camera, website application (development, hosting and maintenance) and training of the GPs and dermatologists. The website is a stand-alone web-based application not integrated with other systems in the GPs' or dermatologists' office.

The costs of the internet connection and computer expenses were not taken into account as almost all GP practices and all dermatologists were already well provided. The yearly costs of investments were calculated from depreciation over three years of the original price. The investment costs were calculated regarding a wide implementation in the Netherlands, for all GPs and dermatologists. We assumed that a GP uses the equipment for 60 patients per year (1,5 per week) [9].

The costs of conventional GP care only consisted of the first consultation costs (duration of consultation with referral to a dermatologist), while teledermatology GP care costs also contained the costs of the diagnostics and treatment following the teledermatology advice, as well as subsequent follow-up visits. The duration of the teledermatology activities in a GP practice was estimated in a separate laboratory study among eight GPs with eight patients each [10].

Costs of diagnostics and treatment were estimated by calculating the average costs of diagnostics and treatment associated with six out of ten diagnoses for which Dutch patients are most often referred to a dermatologist [9]. The other four top ten diagnoses (local redness, local swelling, other skin diseases and other benign skin diseases) were not specified well enough to find out the kind of diagnostic and therapeutic measures they imply. Therefore, the final list of diagnoses included 6 instead of 10 diagnoses. Diagnostics and treatment costs in general practice were estimated for those 6 most frequently observed dermatological diagnoses referred by GPs. In case of teledermatology, costs of diagnostics and treatment were put partially (percentage of preventable consultations) on the GPs side and partially on the dermatologists' side. In case of conventional care, all diagnostic and treatment costs were put on the dermatologists' side. The costs of diagnostics and treatment included only the costs of medication and intervention, while the costs of consultation were separately included in the model.

Two dermatologists provided the following information about each diagnosis: most frequently used diagnostic tests and treatment, in which proportion of the cases they were used and how often a treatment was repeated within a care episode. The dermatological dictionary (http://www.huidziekten.nl, last accessed: October 2007) was used to retrieve further information, verify and complete the list of diagnostic tests and treatments. For each diagnostic test a tariff was used from the Tariff Book of Medical Specialists (http://www.nza.nl, last accessed: October 2007). In order to retrieve the costs of medications, the Pharmaceutical Compass (PhC, Dutch registry of medications) was used. If a drug was not included in the PhC, another drug name, usually the brand name, was searched for on the pharmacy websites to obtain data about their costs. Multiple expert opinions were similar and therefore easily to aggregate by taking the average. Finally, general prevalence data of the six diagnoses were used to derive a weighted average of the use of health care resources.

Costs of dermatology care included consultation costs (first consultation and follow-up), costs of diagnostics and treatment, and the teleconsultation costs during the teledermatology process. The consultation costs were calculated based on the consultation costs per minute including medical staff, hospital housing and overhead [11]. The duration of teleconsultation activities was retrieved from observations of dermatologists during the trial. This information was verified by analysing the logging information from the website application. In 14% of necessary consultations, the dermatologists in our trial indicated that teledermatology shortened the first dermatological consultations remarkably (10%). This finding was incorporated in the model as a variable (distribution, beta integral form: n = 3, r = 30) for the teledermatology consultations that are not prevented. Diagnostic and treatment costs in dermatological practices were estimated following a similar procedure as used in GP practices. For the unit costing of consultations by a dermatologist we distinguished teaching and non-teaching hospitals and calculated a weighted average unit cost given the fact that 10% of Dutch hospitals are teaching hospitals [10].

Out-of-pocket expenses consisted of travel costs of the patient and, if opportune, of an accompanying person. We estimated (based on trial population) that about 20% of patients (children and elderly) visit a health professional in company. Out-of-pocket expenses related to extra, informal help at home were not included because we assumed that they are negligently small in the case of dermatological conditions.

Employer costs originating from production losses at work were derived from the time spent by the patient (GP, dermatologist and pharmacy visit time including travelling, waiting and actual consultation duration) and assuming that the proportions of patients in our sample with a paid job or working part-time resemble national averages.

Unit costs of GP and outpatient dermatological consultations, average travel distances, and national estimates of work participation were derived from the Dutch Manual on Costing in Health Care Research [10].

In case no sources for the unit costs were available (e.g. patient waiting time in the waiting room), expert opinions and educated guesses were used. For website development costs, hosting and maintenance a professional ICT organisation providing such services was consulted.

### Incremental cost analysis and uncertainty analysis

An incremental cost analysis was performed to demonstrate which strategy was associated with the lowest costs per episode of care. Additionally, a probabilistic sensitivity analysis based on a second order Monte Carlo simulation with 10000 simulations was performed to study the impact of parameter uncertainty regarding the estimated unit costs and the volume data on the results of the incremental cost analysis.

A multivariate probabilistic sensitivity analysis was performed with all parameters that, according to one-way sensitivity analysis, influenced model outcome [12]. This analysis resulted in 31 parameters. A linear regression of the cost difference between teledermatology and the conventional process with the sampled distributions as covariates identified the following top five ranking of parameters with the highest impact on the difference (based on the absolute value of the standardized regression coefficients in decreasing order): proportion preventable consultations, number outpatient visits per episode, duration evaluation website teledermatologist, proportion reduce in consultation duration first outpatient consultation, and duration GP follow up consultation.

For all 31 parameters included in this uncertainty analysis, we defined a distribution (range and type) based on observations [10], expert opinion or available resources [11]. The four proportions in the model followed a beta distribution, while a triangular distribution with the most likely value at the top was assumed for other variables (e.g. travel distance). Mean costs of the teledermatology and conventional processes were based on the simulation results.

### Scenario analysis

After checking the face-validity of our findings by presenting it at scientific meetings, we performed a scenario analysis. The proportion of preventable consultations was considered one of the most important variables to decrease the costs due to teledermatology. From our trial, it appeared that the proportion of preventable consultations may vary by disease group. Therefore a range of different proportions of preventable consultations (10% - 50%) was used in one way sensitivity analysis to assess the impact of their variation on changes in costs.

Our study was based on Dutch circumstances where patient distances to GP and dermatologist are relatively small. In order to enable comparisons with other countries, we extended these parameters in a scenario analysis. Furthermore, as there is no standard teledermatology software, time needed to complete teleconsultation activities might vary in different settings. For teledermatology activities on the dermatologist side which were grouped in one variable in the cost model, a one-way sensitivity analysis was applied. In order to assess the impact of a change in teledermatology activities on the GP side, one-way sensitivity analysis was performed using the most consuming GP teleconsultation activity: completing the website forms [10]. In the trial, completing the website forms took on average 3.2 minutes (range: 1.8 to 6.3) [10].

The middle column of Table 1 shows an overview of the main variables considered in the uncertainty and scenario analyses. Data Tree Age Pro Suite 2006 software was used for the calculation of the costs, uncertainty and scenario analyses (DATA Professional/DATA 4.0 Healthcare user's manual. TreeAge Software Inc, 1998-2001).

## Results

### Total costs of teledermatological and conventional processes

**Figure 2:**
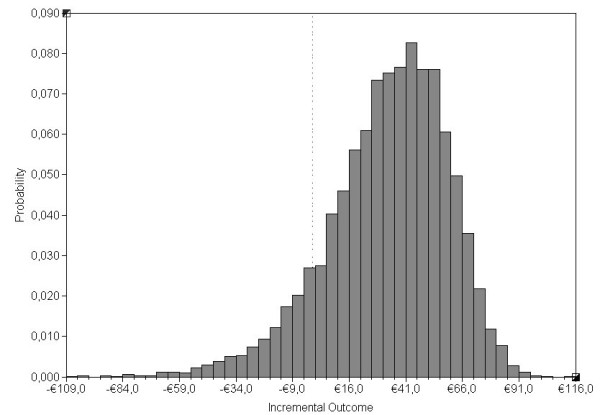
**Distribution of the incremental costs of the teledermatological against conventional process as the reference case, following 10000 Monte Carlo simulations of 31 input distributions**.

Mean costs of the teledermatological process per care episode were €387 (95%CI, 281 to 502.5) and mean conventional process costs were €354.0 (95%CI, 228.0 to 484.0), a total mean difference of €32.5 (95%CI, -29 to 74.7) (Table 2). Monte Carlo simulation showed that the conventional process was less expensive than the teledermatology process in 89% of all simulations (Figure 2).

**Table 2:** Mean costs of teledermatology and conventional process by type of costs, per episode

		Teledermatology process, €	Conventional process, €	Incremental costs due to teledermatology €
Total costs*	Mean total (95% CI)	387.0 (281.0 *to *502.5)	354.0 (228.0 *to *484.0)	32.5 (-29.0 *to *74.7)

Investment costs	Mean total (95% CI)	1.6 (1.6 *to *2.1)	0	1.6 (1.6 *to *2.1)
	
	Digital camera costs	0.8	0	
	
	Website costs	0.16	0	
	
	Training costs	0.6	0	

GP costs	Mean total (95% CI)	85.5 (64.8 *to *111)	21.3 (13.0 *to *30.1)	64.2 (46 *to *88.3)
	
	Costs of diagnosis and treatment	18.4	0	
	
	Costs first visit	45.6	21.3	
	
	Costs follow up visit	17.6	0	

Dermatologist costs	Mean total (95% CI)	241.0 (155.4 *to *338.9)	269.1 (173.8 *to *363.3)	-28.1 (-90.0 *to *15.6)
	
	Costs of diagnosis and treatment	68.9	87.3	
	
	Costs first visit	42.9	54.4	
	
	Costs follow-up visit	48.3	60.4	
	
	Teleconsultation costs	47.5	0	

Out-of-pocket costs	Mean total (95% CI)	12.4 (5.4 *to *19.6)	16.3 (8.1 *to *24.5)	-4.1 (-8.1 *to - *1.85)
	
	Travel costs patient	11.5	15.2	
	
	Travel costs accompanying person	0.8	1.1	

Employer costs	Mean total (95% CI)	46.2 (18.4 *to *86.1)	47.3 (18.0 *to *83.1)	-1.1 (-14.3 *to *7.6)

The 95% confidence interval (95% CI) reported in this table represents the 2.5% and 97.5% percentiles of the simulation results. For interpretation, a Bayesian perspective on uncertainty is adopted in the text of this paper in which the probability of simulations results (e.g. teledermatology being cheaper than conventional practice) is reported.

### Distinct cost components

Mean investment costs were €1.6 per episode and the major part of these costs were the costs of the digital camera. Largest differences in costs were found for the costs of the GP and out-of-pocket costs (Table 2). The GP costs amounted to on average €21.3 euros in the conventional process and €85.5 in the teledermatological process. This difference can be explained by the increased first consultation costs of teledermatology GPs which amounted to €45.6 compared to €21.3 for conventional GPs as the consultations were longer. Furthermore, during this GP consultations in teledermatology process, also costs of diagnostics and treatment are present and there is a follow-up consultation. Mean out-of-pocket costs amounted to €16.3 in the conventional process and €12.4 in the teledermatological process. Prevented outpatient hospital consultations resulted in lower travel costs which were on average €11.5 compared to €15.2 in the conventional process. Dermatologists' costs remained almost the same. The reduction in live consultation lowered the visit costs in the teledermatology process but that was compensated for by the costs of teleconsultation by the dermatologist. There was almost no difference in employer costs, with teledermatology employer costs of €46.2 and €47.3 in the conventional process.

### Scenario analysis

**Figure 3:**
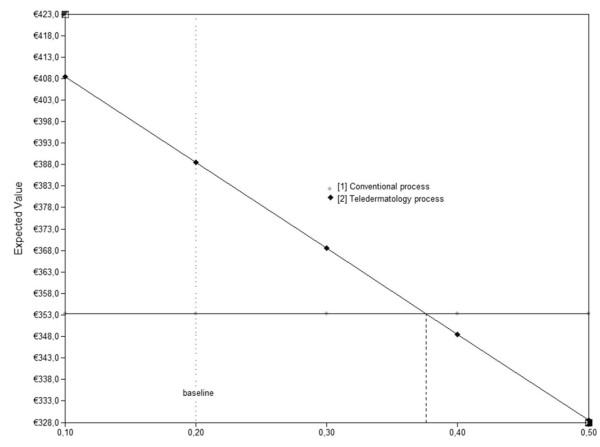
**Total costs of teledermatology care and conventional care (Y-as) for different proportions of consultations prevented (X-as)**.

The scenario-analysis showed that the teledermatology and conventional costs would have been similar (€353.3; see Figure 3), if 37% of referrals by the GPs would have been prevented. Increasing the proportion of preventable consultations decreased the costs of teledermatology care. Observing a proportion of prevented referrals of 37% or higher however, is not that likely (less than 5.5 per 100 simulations).

**Figure 4:**
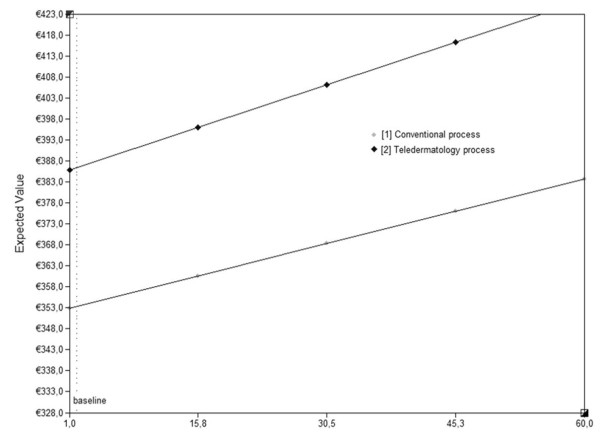
**Total costs of the teledermatological and conventional process (Y-as) for different patient travel distances to GP (X-axis)**.

**Figure 5:**
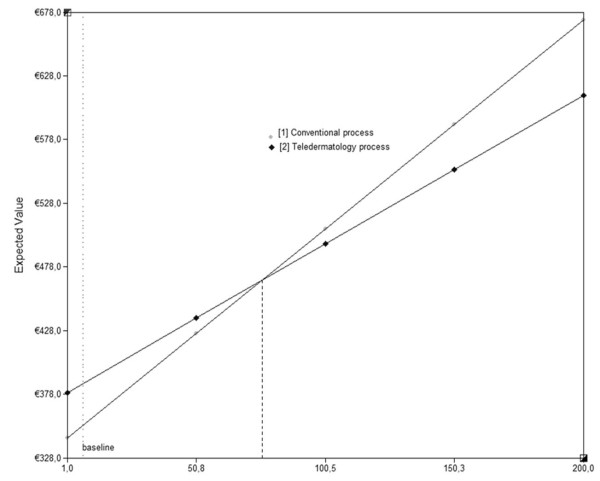
**Total costs of the teledermatological and conventional process (Y-as) for different patient travel distances to dermatologist (X-axis)**.

Extending average patient travel distance to GP (up to 60 km) had a limited impact on the difference in costs, but for the distance to the hospital (up to 200 km) a break even point at 75.1 km was noted. If a dermatologist is this far, which is not very likely in the Netherlands, total costs of both teledermatological and conventional process reach €465.7 (Figure 4 and 5).

Variation in duration of GP teleconsultation regarding completion of the website forms revealed that teledermatology is more expensive if the duration of completing the website forms increases. Change in teleconsultation activities by a dermatologist resulted in the same costs for the two processes at 2.1 minutes. Values up to 2.1 minutes for website completion were observed in less 1.4 per 100 simulations.

## Discussion

Based on our cost model, the economic evaluation showed that the teledermatological process was 33 euro more expensive than the conventional process.

In the Netherlands, as in many other countries, a dermatological consultation is more expensive than a GP consultation. Therefore, shifting the care from a dermatologist to a GP practice would be preferred from an economical point of view. However, when using teledermatology a GP is required to perform extra activities such as obtaining the images and completing the website forms which increases the time of a GP consultation. If a teledermatologist can advise a GP on how to diagnose and/or treat the patient, the costs of diagnostics and treatment are made in the GP practice instead of the dermatologist office. For these reasons, the GP costs of the teledermatology process were higher compared to GP costs in the conventional process. Furthermore, although teledermatology can prevent a fifth of all dermatology referrals [5], this did not result in cheaper total care of dermatology patients as a dermatologist had to review all the patients through teledermatology to finally also see 80% of the patients live.

An important condition for applying cost minimisation analysis (CMA) design is that there is no difference in health outcomes between the two types of care provided [7]. Pak et al. [6] assessed the clinical course of randomly assigned patients to teledermatology or conventional care. As this first large randomized trial confirmed our assumption for equal outcomes between the groups after a certain period, we believe that CMA was an appropriate study approach for the present economic evaluation.

We performed a CMA based on a costs model where a majority of the unit costs were derived from a standard value or were estimated from various resources. In order to verify the robustness of our findings for reasonable alternative parameter estimates, we performed a Monte Carlo simulation. This uncertainty analysis showed that the teledermatological process was more expensive with 89% certainty. One should be aware however, that this percentage is partly based on assumed triangular and beta distributions in the model. Although making such assumptions is common practice, it may lead to over- and underestimations, depending on how well the assumptions meet reality. The model-based approach to assess the costs of teledermatology contains several non-empirical parameter estimates based on expert opinion. Also, data on for example the GP time needed to complete the website were gathered in a convenience subsample of GPs.

Although these estimates may demonstrate face-validity, we accepted broad parameter ranges to account for related uncertainty. Given the limited use of observational data in the present model, the conclusions should be considered as tentative. The results on the relative impact of different parameter estimates may be helpful in designing future studies on the subject.

The proportion of preventable consultations (20%) that resulted from our trial is too low to show a positive cost effect of teledermatology compared to conventional care. When increasing the proportion of preventable consultations up to 40%, teledermatology was more likely to be cheaper than conventional care. In this study we used the dermatologists opinion about preventability of a consultation but in reality it is the GP who decides to refer a patient for consultation. Although we do not have data about the GPs opinion on preventable consultations some studies showed that using teledermatologic consultation in a GP practice with a special interest in dermatology, such as in the United Kingdom [13] might result in a higher proportion of preventable office visits. In our trial, all patients referred to a dermatologist were included. However, the results implicated that the proportion of preventable consultations varied by disease group. Teledermatology appeared to be less suitable for nevi, but very suitable for eczema patients. Regarding this finding, we believe that costs saving due to teledermatology can be reached when teledermatology would be applied in selected groups of patients. However, in order to assess the exact savings for the special groups, a disease-specific trial is necessary and the cost model needs to be adjusted (e.g., costs of diagnosis and treatment).

Extending patient travel distances to a hospital had an impact on the cost difference between the teledermatology and conventional care process. If the distance to a dermatologist is reaching 75 km, teledermatology becomes a cheaper option. For this reason, our study results might be interesting for countries with larger travel distances as in their cases teledermatology might be a cheaper option. However, an adjustment in the cost model would be necessary to assess this assumption (e.g., different care models in different countries, different costs of dermatologists and GPs, different diagnosis and treatment costs).

Scenario analysis showed that the needed time for a teledermatology GP to complete the teleconsultation forms is not important in order to reach savings by teledermatology. In our model, this activity took around three minutes and was almost a third of all teleconsultation activities [10]. Savings due to teledermatology might be reached if the time needed to complete teleconsultation activities by a dermatologist would be reduced to two minutes instead of eight minutes. This duration is however out of range from our observations in the trial, so we consider it unlikely that this scenario would occur.

In this economic evaluation, the costs were calculated per care episode which started with the inclusion of the patient in the trial. The costs before inclusion were not taken into account. It is possible that in some cases a GP tried out a treatment which did not work, and then decided to refer the patient to a dermatologist. In real life GPs would be able to use teledermatology earlier in the care episode than what was prescribed in the context of our trial. Teledermatology potentially lowers the threshold to consult a dermatologist. This can have a different impact on costs than what is demonstrated by this study. The GPs could overuse the service and therefore increase the total societal costs, but it is also possible that there would be less inappropriate treatments in GP practice which would finally lower the societal costs.

Up to our knowledge only one economic evaluation regarding store-and-forward teledermatology based on RCT results has been performed. This study by Whited et al.[14] showed that teledermatology was not cost saving for the healthcare system compared to the conventional care. The researchers indicated that cost savings would be more likely if the societal costs would be considered when teledermatology prevents a third of live consultations. Based on our study results considering societal perspective, we agree with this hypothesis by Whited et al.[14] especially when considering Dutch travel distances. In the past years, several economic evaluations have been performed, especially regarding real-time teledermatology [15-19]. Loane et al. [15] showed a positive effect on costs of real time teledermatology in New Zeeland. In their economic evaluation Armstrong et al.[19] also showed a positive effect on costs by using teledermatology but this study was performed from a healthcare providers' perspective in the United States.

Because of existing differences between the countries in fees, distances and health care systems, our study findings should be generalised with caution. Comparisons with other economic evaluations and their findings should be considered carefully for the same reasons.

## Conclusions

Economic evaluation presented in this paper shows that the chance on societal savings due to the use of teledermatology is relatively small when teledermatology is applied to all dermatology referrals. However, our study shows that savings for the society can be accomplished when teledermatology is applied in countries with larger distances to dermatologists or to specific patient groups where a larger proportion can be treated in a GP practice without the need for a live dermatological consultation.

## Competing interests

The authors declare that they have no competing interests.

## Authors' contributions

NE participated in the study design, acquisition, analysis and interpretation of data, drafting the manuscript, performed statistical analysis, obtained funding, provided administrative support during the trial and had full access to all of the data in the study and take responsibility for the integrity of the data and the accuracy of the data analysis.

MGD participated in the study design, analysis and interpretation of data, performed statistical analysis, obtained funding, supervised the study, contributed to the critical revision of the manuscript for important intellectual content and had full access to all of the data in the study and take responsibility for the integrity of the data and the accuracy of the data analysis. RMB participated in the study design, acquisition of data and contributed to the critical revision of the manuscript for important intellectual content. AH participated in the acquisition, analysis and interpretation of data, contributed to the critical revision of the manuscript for important intellectual content, provided administrative support during the trial and had full access to all of the data in the study and take responsibility for the integrity of the data and the accuracy of the data analysis. PJEB participated in the study design, obtained funding, contributed to the critical revision of the manuscript for important intellectual content and supervised the study.

NFdK participated in the study design, drafting of the manuscript, obtained funding, contributed to the critical revision of the manuscript for important intellectual content and supervised the study. All authors read and approved the final manuscript.

## Pre-publication history

The pre-publication history for this paper can be accessed here:

http://www.biomedcentral.com/1472-6963/10/251/prepub
